# Early Perturbations in Red Blood Cells in Response to Murine Malarial Parasite Infection: Proof-of-Concept ^1^H NMR Metabolomic Study

**DOI:** 10.3390/life13081684

**Published:** 2023-08-04

**Authors:** Arjun Sengupta, Soumita Ghosh, Shobhona Sharma, Haripalsingh M. Sonawat

**Affiliations:** 1 Department of Chemical Sciences, Tata Institute of Fundamental Research, Homi Bhabha Road, Mumbai 400005, India; soumita@pennmedicine.upenn.edu (S.G.); harisonawat@gmail.com (H.M.S.); 2 Department of Biological Sciences, Tata Institute of Fundamental Research, Homi Bhabha Road, Mumbai 400005, India; shobhona@gmail.com

**Keywords:** red blood cell, metabolomics, NMR, malaria, choline, NAD

## Abstract

Background: The major focus of metabolomics research has been confined to the readily available biofluids—urine and blood serum. However, red blood cells (RBCs) are also readily available, and may be a source of a wealth of information on vertebrates. However, the comprehensive metabolomic characterization of RBCs is minimal although they exhibit perturbations in various physiological states. RBCs act as the host of malarial parasites during the symptomatic stage. Thus, understanding the changes in RBC metabolism during infection is crucial for a better understanding of disease progression. Methods: The metabolome of normal RBCs obtained from Swiss mice was investigated using ^1^H NMR spectroscopy. Several 1 and 2-dimensional ^1^H NMR experiments were employed for this purpose. The information from this study was used to investigate the changes in the RBC metabolome during the early stage of infection (~1% infected RBCs) by *Plasmodium bergheii* ANKA. Results: We identified over 40 metabolites in RBCs. Several of these metabolites were quantitated using ^1^H NMR spectroscopy. The results indicate changes in the choline/membrane components and other metabolites during the early stage of malaria. Conclusions: The paper reports the comprehensive characterization of the metabolome of mouse RBCs. Changes during the early stage of malarial infection suggest significant metabolic alteration, even at low parasite content (~1%). General significance: This study should be of use in maximizing the amount of information available from metabolomic experiments on the cellular components of blood. The technique can be directly applied to real-time investigation of infectious diseases that target RBCs.

## 1. Introduction

Metabolomics, since its advent, has been growing as a field that offers immense insights into the biological mechanism of stress response and identification of disease biomarkers. Successful application of this technology has shown encouraging results in real clinical settings, for example, biomarker identification for diabetes [1], hypertension [2], cancer [3], and infectious diseases [4,5,6]. Urine and serum, compared to other biofluids, are more often used for these studies. The reasons for the extensive use of these two biofluids are the easy and non-/minimally invasive nature of sample collection. Urine is an excellent reporter of global metabolic changes in the system and of xenobiotic transformation and excretion by the host. Serum metabolite profiling provides information on homeostatic regulatory functions. The information gathered from blood can be significantly enhanced by profiling cellular components that play a crucial role in the disease process or may be targets of a parasitic infection. Thus, the profiling of RBCs is expected to provide insights into the molecular mechanisms of diseases. As these cells lack subcellular organelles, the metabolic network of mature RBCs is limited to housekeeping functions and diseases that directly involve RBCs are known to alter metabolic pathways therein. Our group is involved in the ^1^H NMR-based metabolomic investigation of various aspects of disease progression in humans and a murine model of malaria. We therefore investigated the metabolome of RBCs. For example, previous studies indicated that hypoxic red blood cells (RBCs) are able to induce vasodilation through the generation of nitric oxide and the release of ATP [7]. Although the metabolic network of RBCs has remained very much confined to the glycolytic pathway and few other pathways because of a lack of cellular organelles, diseases that directly involve RBCs are known to alter the metabolic pathways. Earlier studies found major alterations in the glycolysis pathway during the intraerythrocytic stage of malarial infection [8]. In addition, other metabolites such as glutamine and α-ketoglutarate, which are not directly synthesized in RBCs are evidently present as substrate for RBC metabolic pathway such as glutathione generation [9]. Therefore, it is possible that RBCs can provide useful information if profiled under relevant stress or perturbed conditions. Metabolomic studies of RBCs, although not as extensive as urine and/or serum, do exist. However, almost all are based on mass spectrometry [10,11,12]. However, NMR-based metabolomic methods have the advantages of minimal sample preparation, unbiased nature of the technique, reproducibility, and minimum time requirements. Additionally, the power of this method lies in the quantifiability of metabolites. Furthermore, NMR spectroscopy helps to identify metabolites. Therefore, NMR-based metabolomics remains a much favored technology, despite the complex nature of the spectra and relative insensitivity.

Here, we report the NMR-based spectroscopic characterization of the hydrophilic fraction of RBC using several conventional NMR techniques. We used 1-dimensional, 2-dimensional J-resolved, TOCSY, and HMQC NMR to identify well over 40 metabolites. In addition, distinct peaks from ^1^H NMR spectra were used to quantify 25 of these metabolites. In order to demonstrate the potential of this method, we show the early host metabolic response in the RBCs of Swiss mice infected with *Plasmodium berghei* ANKA. Previous results from our group demonstrated distinct changes in blood serum metabolic profiles in rodent malarial models as well as human subjects [13,14,15,16,17,18]. In early stage of rodent malarial infection, parasite load is similar to that observed in the majority of human infection. Moreover, since the overwhelming majority of RBCs are still uninfected, it may be safe to assume that the observed differences are arising from healthy RBCs. As such, it was reported that glucose utilization is perturbed in the uninfected RBCs from infected animals [8]. Physiological changes during this stage may be able to map the key events of disease progression. We have demonstrated changes in RBC glucose utilization during early infection stage (<1% parasitemia) in the host RBCs in a BALBc/PbANKA model. Specifically, the 2,3-bisphosphoglycerate shunt was found to be reduced [8]. Therefore, we extended the approach to cover more metabolites using the current NMR-based methodology. Our results suggest alterations in the cell membrane lipid components of the RBCs as well as the changes in the redox balance upon parasitic infection.

## 2. Materials and Methods

### 2.1. Animal Experiments

The animals were treated in accordance with the guidelines set forth by the Local Animal Ethics Committee.

### 2.2. Collection, Lysis, and Extraction of Red Blood Cells

In order to collect the RBCs for assessment purposes, six healthy male Swiss mice of 6–8 weeks of age were used. The mice were maintained at 25 ± 2 °C with 12 h day–night cycle and had access to standard food pellets and water ad libitum. The blood samples were collected from these mice using retro-orbital bleeding in microtubes containing a solution of 0.22% EDTA and 0.9% NaCl (both final concentrations). This process generated ~100 μL of blood from each mouse. The collected blood was centrifuged at 600× *g* for 5 min (hematocrit ~0.4). The supernatant was discarded and the pellet was suspended in 400 μL 0.9% NaCl solution and re-centrifuged. The obtained RBC pellet was separated and lysed in distilled water (600 μL) by vortexing and kept at 4 °C for 15 min. Then, 500 μL of methanol and 400 μL chloroform were added to the RBC lysate, which was vortexed and centrifuged at 5000× *g* for 15 min. The methanol–water portion was separated and the solvent was evaporated off by a vacuum concentrator. The residues were pooled by reconstituting in 600 μL D_2_O. Further, 100 μL stock solution of 0.07% TSP (sodium trimethylsilyl propionate, Cambridge Isotope Laboratories, Andover, MA, USA) in D_2_O was added. This sample was directly used for NMR spectroscopy for assignment purpose. D_2_O served as a field-frequency lock and TSP was the reference compound, with its chemical shift assigned as zero.

### 2.3. Parasite Inoculation, RBC Collection, and Extract Preparation from Sets of Infected and Control Mice

Twelve 6–8-week-old male Swiss mice were used for this experiment. Six mice were inoculated intraperitoneally with 10^6^ *Plasmodium berghei* ANKA-infected RBCs maintained in male Swiss mice of the same age. On the second day after infection, after measurement of parasitemia (</~1%) by microscopy of Giemsa-stained blood smears from the infected mice, blood from all 12 mice was drawn using retro-orbital bleeding and the mice were sacrificed. The samples for NMR spectroscopy were prepared as mentioned above. The hematocrit was ~0.4 and did not significantly differ between the infected and uninfected mice. Note that the individual samples were treated as such in this case and were not pooled as described in Section 2.2.

### 2.4. ^1^H NMR Spectroscopy, Assignment, and Quantification of the RBC Metabolome

All the spectra were recorded at 298 K. The 1-dimensional ^1^H NMR spectrum, 2-dimensional J-resolved, and TOCSY spectra were acquired at 298 °K on a Bruker AVANCE-II 700 MHz spectrometer (Bruker Biospin, Karlsruhe, Germany) fitted with a TBI probe. The HMQC spectrum was recorded in a Bruker Avance-II 500 MHz spectrometer fitted with a TXI probe. The 1-dimensional ^1^H spectra were recorded using a pulse program that resembles the 1st increment of the NOESY experiment with gradient pulsing and saturation of water signal during the 4 s relaxation delay. This spectrum was recorded with 64 scans and 32k data points. For processing, the FID was zero-filled to 64k, multiplied by an exponential function leading to an additional line broadening of 0.3 Hz, and Fourier transformed and automatically phase- and baseline-corrected. The J-resolved spectrum was acquired with a 1 s relaxation delay during which the water resonance was saturated. In the direct dimension for each of 16 scans, 8k data points were acquired, and 40 such transients were recorded in the indirect dimension. The FID was multiplied with a sine window function in both dimensions and line-broadening factors of 1 Hz and 0.3 Hz were used in the direct and indirect dimensions, respectively, prior to Fourier transformation. The spectrum was tilted to 45° along F2 and the baseline was corrected automatically.

To record the TOCSY spectrum, a relaxation delay of 1.5 s was used. The transients (150 scans) were stored in 2k data points; 256 such experiments were performed in the indirect dimension. For processing, a sine window function was used in both dimensions, along with a line-broadening factor of 1 Hz and 0.3 Hz along the direct and indirect dimension, respectively. In addition, a linear prediction function with 32 data points was used in the indirect dimension. The spectrum was Fourier transformed, and phase and baseline were corrected manually. An excitation sculpting routine was used for the suppression of water signal. HMQC spectrum was acquired with a 2 s relaxation delay, during which the water signal was saturated. The data (128 scans) were stored in 2k digital points in the direct dimension and 256 such experiments were performed in the indirect dimension. For processing, the QSINE window function was used along with a line-broadening factor of 0.3 Hz in both the dimensions. The FIDs were Fourier transformed and manually phase corrected. The baseline correction was performed automatically.

In order to assign the NMR resonances, the following strategy was adopted. Initially, several metabolites could be directly assigned using the chemical shift values and coupling constants obtained from the 1-dimensional ^1^H NMR spectrum of the sample. For resolving peaks with complex and overlapping multiplet pattern, J-resolved spectrum of the same sample was acquired. These multiplet patterns were further associated with the coupling pattern observed in the 2-dimensional TOCSY experiment. For assigning the singlets seen in the J-resolved as well as the 1-dimensional spectral profile, the 2-dimensional ^1^H-^13^C HMQC spectrum was acquired. All the correlations and spectral patterns observed were further confirmed by database searches (www.hmdb.ca, accessed on 31 August, 2013 and http://mmcd.nmrfam.wisc.edu, accessed on 31 August 2013) and earlier literature [13].

In order to have an estimate of the concentrations of the metabolites in RBC samples, the 1-dimensional ^1^H NMR spectra of the uninfected control mice were used. The peaks that could be distinctly identified in the spectra were integrated and normalized to the TSP signal intensity. The concentrations of the metabolites were then calculated using the following formula.

Ca = (Ia/Na)*(Ns/Is)*Cs

where Ca = concentration of analyte, Cs = concentration of reference (TSP), Ia = Integral (area) of the analyte peak, Is = Integral (area) of the TSP peak, Na = Number of nuclei contributing to the analyte signal, Ns = Number of nuclei contributing to the TSP signal.

To investigate the changes in the RBC metabolic profile during malarial infection, the RBC profiles of six uninfected and six infected mice were acquired with 1-dimensional ^1^H NMR, as mentioned earlier, using 1k scans.

### 2.5. Multivariate and Univariate Data Analysis and Quantification of Metabolites

In order to delineate the RBC metabolic response during early stage of malarial infection, the 1H NMR spectra from the 12 mice were imported to AMIX v3.8.4 (Bruker Biospin, Karlsruhe, Germany), binned into 0.04 ppm frequency buckets, integrated, and normalized to the whole spectral window. The generated data matrix was imported into SIMCA P+ 12.0 (Umetrics Inc., Umeaa, Sweden), mean centered, and Pareto scaled. Principal component analysis (PCA) was performed on this data matrix. PCA reduces the multidimensional NMR data to a smaller number of principal components (PCs) according to the variation in the data set. The samples could therefore be projected on the new PC space to identify the pattern in the sample set, which may be visualized by the scores plot. Each PC is a linear combination of the original variables and the weight of each variable on a certain PC could be identified by a loadings plot.

NMR peaks in spectral bins found to vary significantly between the infected and uninfected animals (as described in Section 2.3) were identified in the original NMR spectra and integrated and normalized to the total spectral intensity using AMIX. They were further compared using Student’s *t*-test.

To determine the concentrations of the metabolites in the RBC samples, the 1-dimensional ^1^H NMR spectra of the uninfected control mice were used. The peaks that could be distinctly identified in the spectra were integrated and normalized to the TSP signal intensity.

## 3. Results

### 3.1. ^1^H NMR Spectroscopy of the RBC Lysate Extract, Assignments, and Quantification of the Metabolites

**Figure 1:**
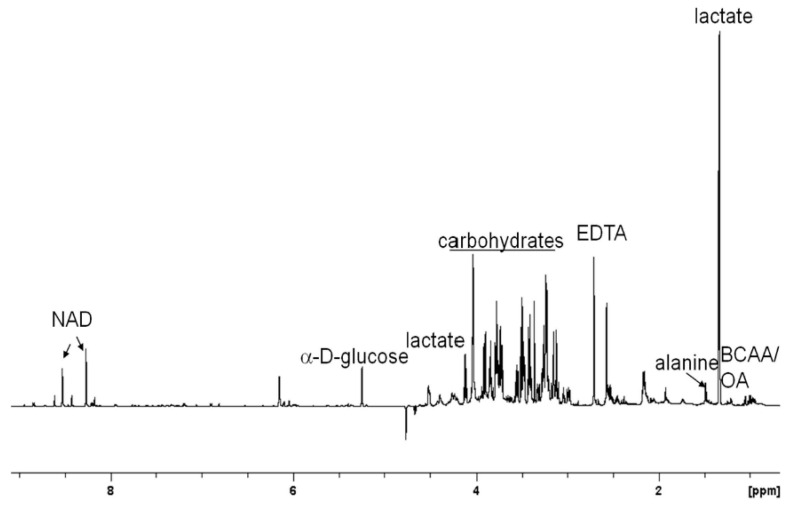
The 700 MHz 1-dimensional ^1^H NMR spectral profile of the water–methanol fraction of pooled RBC lysate extract from six 6–8-week-old male Swiss mice. Only the distinct metabolites/classes of metabolites are assigned here. Further assignments are achieved using a combination of 2-dimensional NMR spectroscopic techniques. Keys: BCAA/OA—branched chain amino acids and organic acids, NAD—nicotinamide adenine dinucleotide.

Several metabolites could be identified from the 1-dimensional ^1^H NMR spectrum of the pooled RBC lysate extract (Figure 1). For example, peaks from lactic acid (1.33 ppm, d and 4.12 ppm, q), branched chain amino acids (valine, leucine, and isoleucine, 0.95–1.04 ppm, m) and alanine (1.48 ppm, d). In addition, several multiplets from carbohydrates and α-^1^H of amino acids could be observed in the region of 3.25 to 4.00 ppm. Several signals from the aromatic region were also present. However, as a relatively unexplored system, these peaks could not be identified directly from the 1-dimensional spectrum.

**Figure 2:**
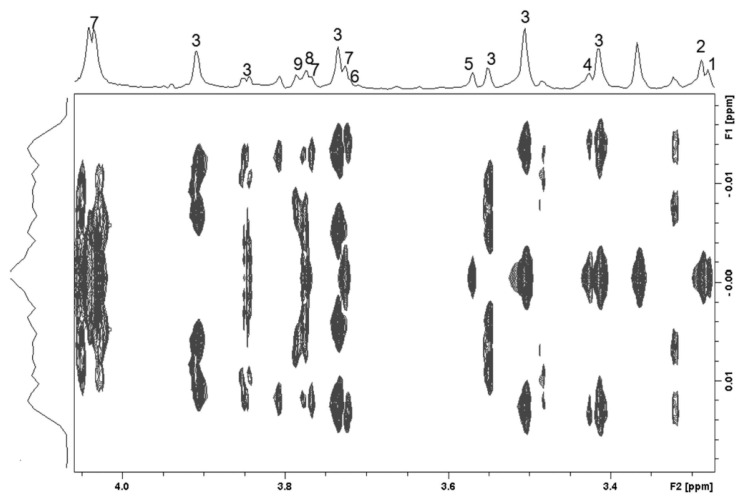
The 700 MHz 2-dimensional ^1^H J-resolved NMR spectral profile of the water–methanol fraction of pooled RBC lysate extract from 6–8-week-old male Swiss mice. Only the complex regions of 3.2–4.1 ppm are presented. Further assignments are achieved using a combination of 2-dimensional NMR spectroscopic techniques. Keys: 1—betaine, 2—TMAO, 3—glucose, 4—taurine, 5—glycine, 6—dimethylglycine, 7—ascorbate, 8—guanidoacetate, 9—erythritol. The multiplicities and chemical shifts of the metabolites are put together in Table 1.

**Figure 3:**
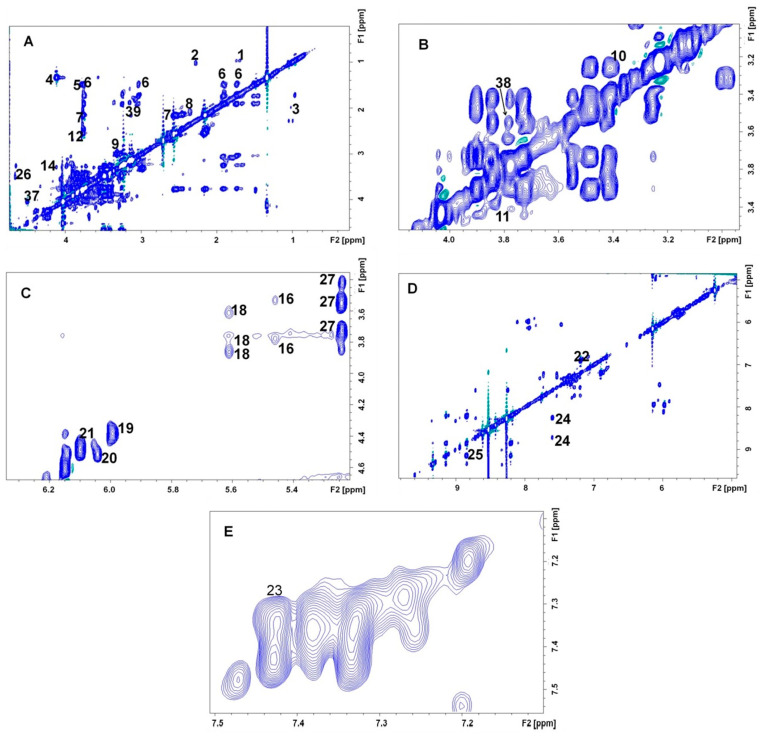
Annotated regions of 700 MHz ^1^H-^1^H TOCSY experiment from pooled RBC lysate extract from 6–8-week-old male Swiss mice. (**A**) The aliphatic region of the spectrum, (**B**) the crowded region containing carbohydrate signals, (**C**) signals from the α-^1^H of carbohydrate-like metabolites, (**D**) aromatic region of the spectrum, (**E**) peaks close to diagonal from the aromatic region. Keys: 1—leucine, 2—valine, 3—isoleucine, 4—lactate, 5—alanine, 6—lysine, 7—glutamine, 8—glutamate, 9—histamine, 10—taurine, 11—serine, 12—aspartate, 14—proline, 15—pentose ring of NAD/NADH, 16—glucosamine, 17—NADH, 18—UDP-glucose, 19—uridine-5′ monophosphate, 20—AMP, 21—ATP, 22—tyrosine, 23—phenylalanine, 24—niacinamide, 25—NAD, 26—b-D-glucose, 27—a-D-glucose, 37—ascorbate, 38—glycerol, 39—citrulline. The multiplicities and chemical shifts of the metabolites are put together in Table 1.

The 2-dimensional J-resolved spectrum (Figure 2) helped in identification of several multiplet structures in the crowded region (3.25–4.00 ppm) of the spectral profile. Thus, singlets from several choline derivatives, carbohydrates, and taurine could be identified. The assignments were corroborated by the coupling patterns from the TOCSY spectrum (Figure 3) and several multiplets could be assigned unequivocally. For example, distinct peaks were observed for each of the branched chain amino acids (leucine—0.95, 1.69 ppm; valine—0.99, 2.27, 3.60 ppm, and isoleucine—1.01, 1.98 ppm). Peaks and coupling patterns from other amino acids such as lysine (1.46, 1.51, 1.74, 1.91, 3.04, 3.76, ppm), glutamine (2.16, 2.55, 3.77 ppm), glutamate (2.10, 2.35, 3.76ppm), histamine (2.98, 3.29 ppm), citrulline (1.56, 1.87, 3.14 ppm), serine (3.84, 3.96 ppm), aspartate (2.68, 3.91 ppm), and proline (3.28, 4.13 ppm) were identified. Signals from certain aromatic amino acids including tyrosine (6.90, 7.19 ppm) and phenylalanine (7.34, 7.43 ppm) could also be ascertained in the downfield region of the TOCSY spectrum. Carbohydrates and their derivatives were also distinguishable from the TOCSY profile. α- and β-D-glucose were identified separately. Among other carbohydrates, we could assign erythritol (3.66, 3.78 ppm), UDP-glucose (3.60, 3.75, 3.86, 5.60 ppm), and D-glucosamine (3.53, 3.77, 5.46 ppm).

**Figure 4:**
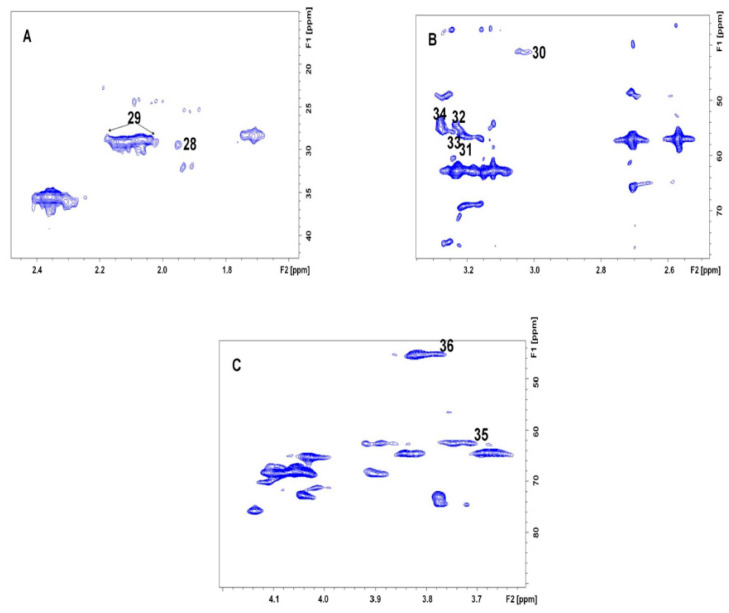
(**A**–**C**)—Various segments of the 500 MHz HMQC spectrum acquired from pooled RBC lysate extract from 6–8-week-old male Swiss mice. Keys: 28—acetate, 29—N-acetylated groups of various metabolites, 30—creatine, 31—choline, 32—phosphorylcholine, 33—glycerophosphorylcholine, 34—betaine, 35—dimethylglycine, 36—guanidoacetate.

However, the identification of metabolites with singlet peaks was not possible using the TOCSY spectrum. Therefore, the ^1^H-^13^C HMQC spectrum (Figure 4) was acquired for the pooled sample. Several singlet peaks were identified by this procedure. Thus, choline (3.21–56.38 ppm) and its derivatives, phosphorylcholine (3.22–54.3 ppm) and glycerophosphorylcholine (3.24–55.68 ppm), were observed on the basis of the –N^+^(CH_3_)_3_ groups. Similar signatures helped us to assign trimethylamineoxide (3.29–61.34 ppm) and betaine (3.28–54.40 ppm). Additionally, metabolites such as acetate (1.95–29.3 ppm) and metabolites containing N-acetylated groups ((2.02–2.06)–(28.8–29.07) ppm) were identified. A comprehensive list of the identified metabolites is presented in Table 1. NMR coupling patterns (either from TOCSY or HMQC) were detected for nine additional metabolites. However, these metabolites could not be identified (Supplementary Table S1).

For the quantitation of the metabolites, the distinct peaks observed in the 1-dimensional ^1^H NMR spectrum of the samples from the six control mice were integrated with respect to the TSP signal (Supplementary Table S2). Of the metabolites that were present at a high level, the concentration of lactate was ~4.7 mM and that of glucose was ~1.7 mM. Among the amino acids, the glutamine concentration was ~1.8 mM. Several other amino acids, at concentrations of ~100 μM, could also be observed. In addition, several other metabolites with concentrations of tens of μM were also quantifiable.

### 3.2. Metabolic Response of RBC during Early Stage Malarial Infection

**Figure 5:**
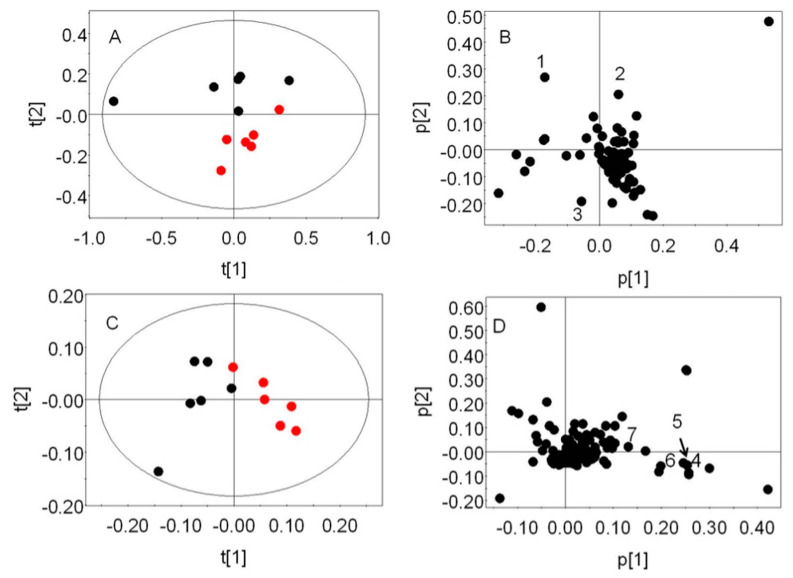
PCA scores (**A**,**C**) and loadings (**B**,**D**) plots from the 1-dimensional ^1^H NMR spectral profile of the RBC lysate extract from 6 -8-week-old male uninfected Swiss mice (red in the scores plots—**A**,**C**) and infected with *Pb*ANKA (black). The PCA models were generated by treating the aliphatic region (0.5- 4.5 ppm) and the downfield aliphatic and aromatic region (5.1- 9.5 ppm) separately. The segregation was seen in PC2 (R^2^X(2) = 0.18) and PC1 (R^2^X(1) = 0.51), respectively. Keys from the loading plots: 1—GPC; 2—PC; 3—ascorbate and glycerol; 4—NAD; 5—AMP; 6, 7—ATP.

PCA was carried out on the 1-dimensional ^1^H NMR spectra of uninfected male mice and those infected with *P. berghei* ANKA (Figure 5). The purpose of this analysis was to evaluate whether the infection imparts major variation in the global RBC metabolic profile. An early time point, when ~1% of RBCs were infected (therefore, ~99% remained uninfected), was considered. PCA was performed independently on two annotated regions, the aliphatic (0.5–4.5 ppm) and the aromatic (5.1–9.5 ppm) regions. The regions containing the EDTA peaks (2.5–2.8 ppm) were excluded from the analysis. This approach ensured that the comparatively less intense aromatic peaks were not ignored and more information could be extracted from the PCA. The cumulative R^2^X for the first two PCs was found to be 0.87 in the first model (Figure 5A). PC1 was seen to clearly distinguish the one animal from which the total blood volume and, therefore lower packed cell volume, could be recovered. The S/N ratio of the corresponding ^1^H NMR spectrum was poorer in comparison to the other spectra. PC2 (~18% of total variation) showed a distinction between the infected and uninfected animals. The second PCA model that represented the aromatic and downfield aliphatic region of the spectra resulted in a cumulative R^2^X value of 0.88 (Figure 5C). The control and infected animals were clearly segregated along PC1, which explained ~51% of the total variation. The spectral regions that contributed to this segregation were integrated and compared. Significant variation was observed in the levels of glycerophosphorylcholine (GPC), ascorbate, glycerol, phosphorylcholine (PC), proline, NAD, AMP, and ATP. Of these, RBCs of infected animals showed elevated levels of GPC (~1.6 fold) and PC (~3 fold), while ascorbate (~0.9 fold), glycerol (~0.76 fold), proline (~0.56 fold), NAD (~0.7 fold), AMP (~0.5 fold), and ATP (~0.5 fold) levels were low (Supplementary Table S3 and Figure S4). Supplementary Table S5 provides the CheBI IDs of the metabolites reported to vary significantly.

## 4. Discussion

Metabolomics is useful for extracting information on the biological stress response in terms of the level of metabolites. To apply this approach in the field of medical diagnostics and intervention, it is required that the sample be obtained in a process that is as minimally invasive as possible. Thus, metabolite analysis in biofluids has gained traction in recent decades [19]. Urine [20] and serum [21] are the most commonly used samples. Studies using saliva [22] and exhaled breath condensate [23] have also been reported. However, efforts should be made to maximize the cohort of information within the limitation of the ease of sample collection. We believe that the whole-blood metabolome may provide further insights into specific stress responses in addition to those provided by the analysis of serum. RBCs can be separated and extracted with ease by established protocols. However, the number of metabolomic studies using RBCs remains surprisingly small [24]. Here, we present an exploratory investigation of RBC metabolome using NMR spectroscopy.

We used conventional NMR spectroscopic techniques to identify several metabolites in the RBC lysate extracts from Swiss mice. As the metabolic activity of RBCs is limited to glycolysis, the pentose phosphate pathway, and some of the pathways related to housekeeping [25], the metabolome is not expected to be as diverse as that of serum or urine. We identified 43 metabolites; 9 NMR-visible metabolites remained unidentified. Glucose and lactate were found to be the most abundant. Lactate in RBCs is known to be accumulated during the reduction of the glycolytic end product, pyruvate [8]. However, we did not observe pyruvate in the system. The rapid conversion of pyruvate to lactate may render its steady state concentration below the NMR detection limit. The other glucose utilization pathway, i.e., the pentose phosphate pathway (PPP/hexose monophosphate shunt), results in 10% of the total glucose flux. This leads to pentose sugars and glycolytic intermediates such as glyceraldehyde-3-phosphate and fructose-6-phosphate. We did not observe any of the pentose sugars in our study. However, our observations confirmed several compounds containing pentose rings, including NAD, NADH, UMP and UDP-glucose. These metabolites may arise from PPP. We observed several amino acids in our study. Thus, all the branched chain amino acids—lysine, alanine, glutamine, and glutamate—were present. RBCs do not have active amino acid metabolism pathways. However, transport pathways for specific amino acids have been reported in RBCs [26]. These amino acids play specific roles in maintaining the general physiology of RBCs. Thus, glutamate is known to be involved in the synthesis of glutathione, a molecule that RBCs require to maintain redox balance. However, the RBC membrane is almost impermeable to glutamate. Glutamine, in contrast, can cross this barrier and generate glutamate and glutathione [9]. The other amino acids occur in RBCs either via in-transport from the plasma, or from protein breakdown. Mammalian RBCs are known to contain protein degradation pathways [27,28]. However, we also observed the presence of citrulline in the RBC metabolome. Citrulline is not incorporated in the protein; therefore, it is unlikely to originate from protein degradation. The major metabolic pathway giving rise to citrulline is the urea cycle, which does not occur in RBCs. However, the existence of citrulline in RBCs has been reported [29]. This metabolite owes its existence to the action of nitric oxide synthase (NOS) on arginine in RBCs [30,31]. Therefore, the level of citrulline may be used as an indicator of NOS activity in RBCs. In our analysis, we observed certain phosphorylated compounds. In addition to UDP-glucose, UMP, etc., we could also assign AMP and ATP. ATP is generated in the glycolytic pathway. However, we believe that more careful experimental design is needed before using the levels of this molecule for any targeted purpose, as they are known to be sensitive to slight changes in external parameters such as temperature and pH.

As a possible scope of the study, we report the changes in the RBC metabolome during early stage malarial infection in male Swiss mice. Since the extent of parasitemia in our investigation is low (</~1%), the observations may represent a global change in the RBC metabolome rather than being ascribed to alterations in parasitized or non-parasitized RBCs. We observed the elevation of GPC and PC in the RBCs of infected animals during the early stages of infection. The parasites are known to incorporate the host lipids to build the cell membrane, placing significant stress on the choline components [32,33]. This may explain the observed changes in the choline derivatives and may also be related to the reduction in glycerol. The alterations in lipid parameters in the serum in malaria-infected mice have been investigated by our group in earlier studies [34]. However, a detailed mechanistic interpretation will require the investigation of the hydrophobic fraction of the cellular extracts. In addition, we observe a lowering in the level of NAD in RBCs from infected mice. It is plausible that the NAD synthetic pathway [35] is affected by the presence of the parasite. In fact, NAD synthesis and its level is elevated in the *P. falciparum*-infected RBCs [35]. Hence, the suppression of this pathway in the uninfected RBCs probably acts as a response to the infection, at least during the early stages. Detailed mechanistic study of a time course of malaria disease progression using RBC metabolomics may help achieve better understanding of the differentially altered pathways, allowing the identification of potential drug target(s) for the disease.

## 5. Limitations

The study generates intriguing information regarding the metabolic changes in the host RBCs during the early stages of malarial infection. Nevertheless, there are some obvious limitations of this study. Some of the observations in this work need further validation. For example, the impact of changes in the choline components on the RBC lipidome can be further investigated. Additionally, although we detected 40 metabolites and quantified majority of them using a peak integration method, other commercially/openly available deconvolution methods could potentially identify and quantify larger number of metabolites. As such, this approach provides a middle ground between frequently used but error-prone binning strategies and relatively computationally intensive deconvolution methods. Finally, we assumed that the observation of host RBC effects was arising from uninfected RBC popultionas very tiny fraction of total RBCs is infected. However, our current study design is not equipped to make that conclusion beyond all doubt and more careful studies examining the separation of infected and non-infected RBCs are needed to verify this hypothesis.

## Figures and Tables

**Table 1:** The list of identified metabolites from the NMR spectral profile of the pooled sample of RBC lysate extracts (the methanol–water fraction). The ^1^H-^1^H coupling pattern of the metabolites that were identified using TOCSY are provided. The singlet peaks were identified using HMQC experiments and the corresponding ^1^H-^13^C coupling pattern is also provided. The references for the assignments are provided in the text. Peaks highlighted in bold were used for quantification purposes (refer to Supplementary Table S2).

Metabolite	NMR Peak (Multiplicity, Coupling Constant)	TOCSY Pattern	HMQC Pattern
2,3-Dihydroxyvaleric acid	**0.93** (d)	0.94–1.47–3.79	-
Leucine	**0.95** (d, 6.09),	0.95–1.69	-
Valine	0.99(d, 6.9) **1.04** (d, 7.27)	0.99–2.27–3.60 1.04–2.27	-
Isoleucine	**1.01** (d, 7.23)	1.01–1.98	-
3-Hydroxybutyrate	**1.20** (d, 5.78)	1.20–2.31–2.41	-
Lactate	**1.33** (d, 6.76) 4.12(q)	1.33–4.12	-
Alanine	**1.48** (d, 7.25)	1.48–3.79	-
Lysine	1.74 (m) 1.91 (m) **3.04** (m)	1.74–1.91–3.04–3.76–1.51–1.46	-
Acetate	**1.95** (s)		1.95–29.3
N-acetyl groups	2.02 (s),2.06 (s)		(2.02–2.06)–(28.8–29.07)
Glutamate	2.10 (m) **2.35** (m)	2.10–2.35–3.76	
Glutamine	2.16 (m) **2.55** (m)	2.16–2.55–3.77	
Histamine	**2.98** (dd) 3.29 (dd)	2.98–3.29	
Creatine	**3.04** (s)		3.04–41.2
Citrulline	3.13 (m)	3.14–1.87–1.56	
Choline	**3.21** (s)		3.21–56.38
Phosphorylcholine	**3.22** (s)		3.22–54.3
Glycerophosphocholine	3.24 (s)		3.24–55.68
TMAO	3.29 (s)		3.29–61.34
Betaine	3.28 (s)		3.28–54.40
Glucose	3.41 (t, 9.21) 3.48 (m) 3.50 (t, 9.31) 3.55 (dd) 3.73 (dd) 3.84 (m) 3.91 (dd) 4.66 (d) **5.23** (d)		
Taurine	3.42 (t, 9.61)	3.42–3.25	
Glycine	**3.56** (s)		
Erythritol	3.66 (dd) 3.78 (d)	3.66–3.78	
Dimethylglycine	3.72 (s)		3.72–62.51
Ascorbate	3.72 (d, 20) 3.77 (d, 7.35) 4.03 (m) 4.50 (m)		
Guanidoacetate	3.77 (s)		3.77–45.09
Glycerol	3.77 (m)		
Serine	3.84 (m)	3.84–3.96	
Aspartate	3.91 (m)	3.91–2.68	
Proline	4.13 (m)	4.13–3.28	
NAD/NADH (pentose ring)	4.39 (m)	4.39–4.24	
D-glucosamine	5.46 (dd)	5.46–3.77–3.53	
NADH	5.94	5.94–4.76	
UDP-glucose	5.60 (m) 7.95 (d, 7.32)	5.60–3.86–3.75–3.60	
Uridine-5′-monophosphate	5.98 (m)	5.99- 4.38	
AMP	**6.04** (d, 6.14)	6.04–4.76–4.51	
ATP	6.15 (d, 5.92) **8.26** (s) 8.54 (s)	6.15–4.78–4.61	
Fumarate	6.92 (s)		
Tyrosine	**6.94** (d, 8.47)	6.90–7.19	
Phenylalanine	**7.34** (m)	7.34–7.43	
Niacinamide	7.61 (m) **8.70** (d)	7.61–8.24–8.70	
NAD	8.20 (m) **8.84** (d, 4.47) 9.14 (d, 6.45)	8.20–8.84–9.14	

## Data Availability

Data is available on request from corresponding author.

## Supplementary Materials

The following supporting information can be downloaded at: https://www.mdpi.com/article/10.3390/life13081684/s1. Table S1: The list of unidentified metabolites. These are the metabolites for which NMR peaks in the 1- dimensional 1H NMR spectrum and/or correlation pattern in the TOCSY or HMQC spectrum could be identified. However, no metabolite could be annotated. Table S2: Quantification of fraction of metabolites identified in the methanol water fraction of extracted RBC lysate from RBC samples of 6-8 weeks old uninfected male swiss mice (n = 6). Only those metabolites are identified whose peaks were distinctly visible in the 1-dimensional 1H NMR profile. For the NMR peaks, refer to table 1. Table S3: Significantly altered metabolites during early stages of malarial infection. These metabolites were obtained after two separate PCA analysis was performed on the 1H NMR spectral profile of the methanol water fraction of RBC lysate extracts obtained from 6 male Swiss mice (6–8 weeks old) infected with PbANKA and 6 uninfected age and sex matched control animals. The two PCA models were generated by treating the aliphatic region (0.5–4.5 ppm) and the downfield aliphatic and aromatic regions (5.1–9.5 ppm) separately. # *p* < 0.0001, * *p* < 0.05 as observed during univariate analysis. Figure S1: RBC metabolites with strong variance between control and infected animals determiuned by PCA analysis. Y axis represents concentration normalized to total spectral intensity. Table S4: The CheBI IDs of the metabolites varying significantly across the RBCs of mice infected with malarial parasite and uninfected control mice.
