# Supplementary material for: Early Perturbations in Red Blood Cells in Response to Murine Malarial Parasite Infection: Proof-of-Concept 1H NMR Metabolomic Study
Source: Life (Basel). 2023 Aug 4;13(8):1684. doi: 10.3390/life13081684 (PMC10455252; doi:10.3390/life13081684)
Supplement: Supplementary file 1 [file life-13-01684-s001.zip › life-2457446-supplementary.pdf]

Table S1. The list of unidentified metabolites. These are the metabolites for which NMR peaks in the 1- dimensional ^{1}H NMR spectrum and/or correlation pattern in the TOCSY or HMQC spectrum could be identified. However, no metabolite could be annotated.

NMR peak (multiplicity, coupling constant)	TOCSY pattern	HSQC pattern
1.07 (d,7.29)		-
1.14 (d, 7.25)		-
2.88(s)		2.88- 57
3.80 (d, 18)		-
3.85(d, 16.71)		-
4.61(m)	4.61-4.44	-
5.97(d,8.5)		
6.84(s)		
8.52(m)		

Table S2. Quantification of fraction of metabolites identified in the methanol water fraction of extracted RBC lysate from RBC samples of 6-8 weeks old uninfected male swiss mice (n = 6). Only those metabolites are identified whose peaks were distinctly visible in the 1-dimensional ^{1}H NMR profile. For the NMR peaks, refer to table 1.

Metabolites	Median concentration (μM)	Standard error
2,3-dihydroxyvalerate	40.15	3.39
Leucine	117.69	10.70
Valine	98.97	9.97
Isoleucine	50.51	4.28
3-Hydroxybutyrate	34.39	3.84
Lactate	4712.78	645.84
Alanine	250.478	16.41
Lysine	421.67	26.67
Acetate	36.63	2.36
Glutamate	187.97	14.15
Glutamine	1843.19	86.19
Histamine	610.45	35.63
Creatine	85.87	4.41
Choline	42.04	1.93
Phosphocholine	26.33	1.54
Glycine	181.24	13.28
Glucose	1680.67	73.78
AMP	144.48	17.43
ATP	453.68	23.90
Tyrosine	33.37	3.80
Phenylalanine	37.89	3.32
Niacinamide	43.93	8.60
NAD	159.37	21.89

Table S3. Significantly altered metabolites during early stages of malarial infection. These metabolites were obtained after two separate PCA analysis was performed on the ^{1}H NMR spectral profile of the methanol water fraction of RBC lysate extracts obtained from 6 male Swiss mice (6-8 weeks old) infected with *Pb*ANKA and 6 uninfected age and sex matched control animals. The two PCA models were generated by treating the aliphatic region (0.5-4.5ppm) and the downfield aliphatic and aromatic regions (5.1-9.5ppm) separately. # $p<0.0001$, *$p<0.05$ as observed during univariate analysis.

GPC$^{\#}$	Ascorbate*
PC*	Glycerol*
	Proline*
	NAD*
	AMP*
	ATP*

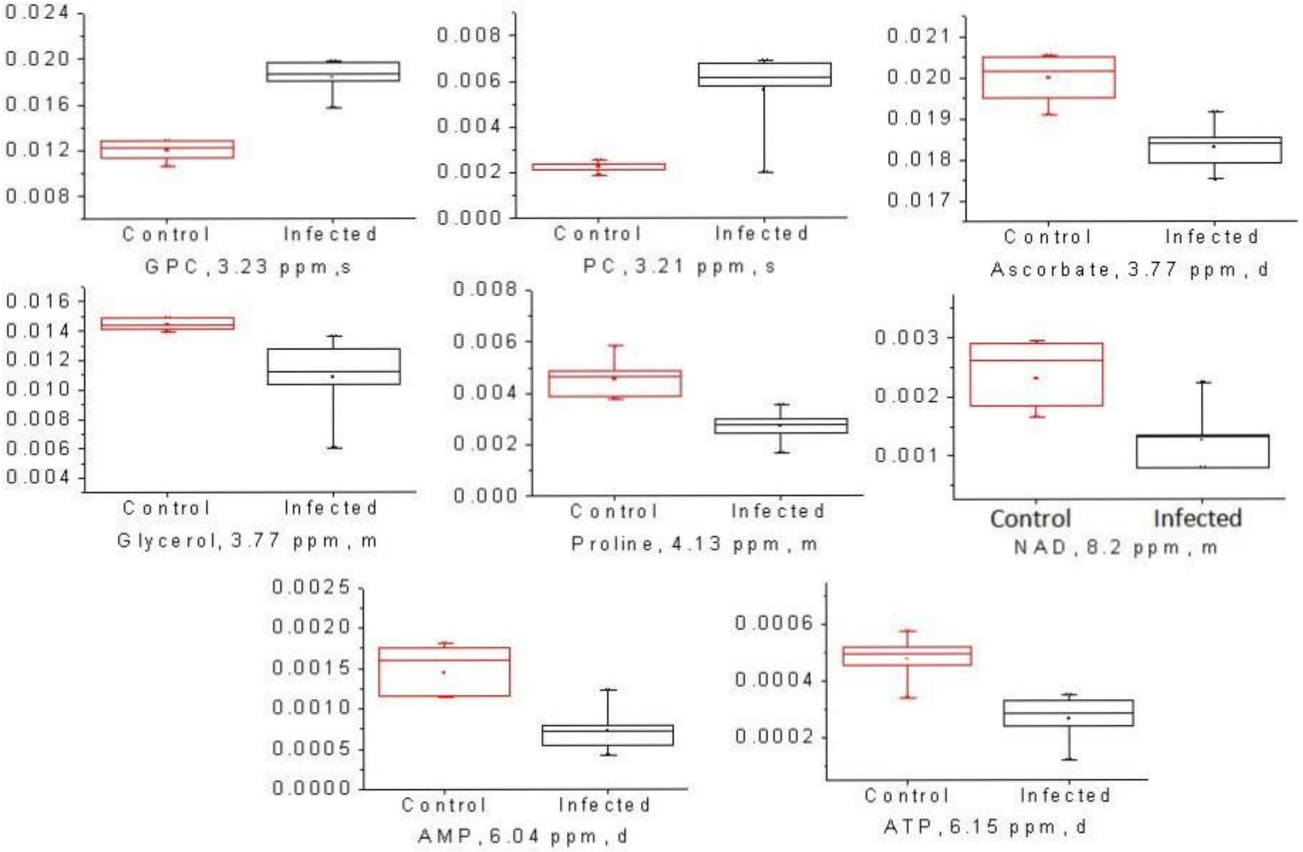

Figure S1. RBC metabolites with strong variance between control and infected animals determiuned by PCA analysis. Y axis represents concentration normalized to total spectral intensity.

Table S4. The CheBI IDs of the metabolites varying significantly across the RBCs of mice infected with malarial parasite and uninfected control mice.

Metabolite	CheBI ID
GPC	36313
PC	18132
Ascorbate	38290
Glycerol	17754
Proline	17203
NAD	13389
ATP	15422
AMP	16027
